# Mechanistic convergence of *Astragalus*-derived phytochemicals in Alzheimer’s disease: A systematic review

**DOI:** 10.1016/j.ibneur.2026.07.015

**Published:** 2026-07-31

**Authors:** Yashvi Bhatt

**Affiliations:** Saint Xavier's College, India

**Keywords:** Astragalus spp., Alzheimer’s disease, Phytochemicals, Herbal medicine, Neuroinflammation

## Abstract

Alzheimer’s disease (AD) is a progressive neurodegenerative disorder characterized by cognitive decline, amyloid-β aggregation, tau pathology, oxidative stress, and neuroinflammation. Despite extensive research, effective disease-modifying therapies remain limited, underscoring the need for multi-target therapeutic strategies. Species of the genus *Astragalus*, widely used in traditional medicine, have gained attention for their neuroprotective potential due to diverse bioactive constituents, including saponins, flavonoids, and polysaccharides. This systematic review synthesizes current preclinical and emerging clinical evidence on the neuroprotective mechanisms of *Astragalus* species in AD. Literature was systematically analyzed in accordance with PRISMA guidelines. The findings indicate that *Astragalus*-derived compounds exert multi-target effects through modulation of key signaling pathways, including PI3K/Akt, Nrf2/ARE, MAPK, and NF-κB. These interactions collectively reduce oxidative stress, attenuate neuroinflammation, inhibit neuronal apoptosis, and improve synaptic function. Several studies further suggest roles in mitigating amyloid-β toxicity and tau hyperphosphorylation. Collectively, the evidence supports a systems-level pharmacological model in which *Astragalus* species mediate convergent neuroprotective effects across interconnected molecular pathways. However, translation to clinical application remains limited by insufficient human studies, variability in species-specific phytochemical profiles, and lack of standardized formulations. Future research should prioritize well-designed clinical trials, comparative species-level analyses, and mechanistic validation to establish the therapeutic potential of *Astragalus* in AD.

## Highlights

• *Astragalus* phytochemicals exhibit multi-target neuroprotection in Alzheimer’s disease.
• Astragaloside IV and formononetin modulate PI3K/Akt, Nrf2/HO-1, and NF-κB pathways.
• Convergent regulation of oxidative stress, neuroinflammation, and apoptosis.
• Species-level phytochemical variability limits translational interpretation.
• Standardization and clinical validation remain key priorities.

## Graphical Abstract

**Figure:**
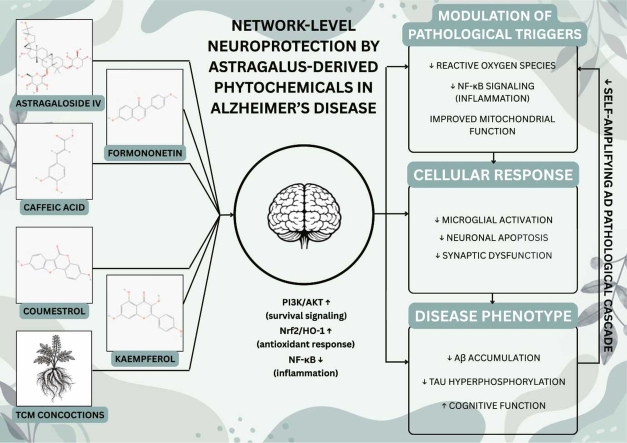

## 1 Introduction

Alzheimer’s Disease (AD) represents a systems-level failure of interacting neurobiological pathways rather than a single-target pathology. Its neuropathology is defined by extracellular β-amyloid (Aβ) plaques, intracellular neurofibrillary tangles (formed by hyperphosphorylated tau proteins), and widespread neuroinflammation (Garbuz et al., 2021). Despite decades of efforts, the currently approved therapeutics such as donepezil, rivastigmine, and memantine only provide symptomatic relief without reversing or even halting neurodegeneration. The complexity of AD pathogenesis, involving an array of neurophysiological disturbances including but not limited to oxidative stress (Chinopoulos and Adam-Vizi, 2006), mitochondrial dysfunction, and iron dyshomeostasis and subsequent ferroptosis (Ghaderi et al., 2024) highlights the need for multi-target therapeutic strategies.

Because the included studies investigated extracts and phytochemicals derived from multiple *Astragalus spp.*, this review uses the general term *Astragalus spp.* except where a study specifies a particular species.

*Astragalus spp.* has traditionally been prescribed for fatigue, immune weakness, and age-associated cognitive decline, and is frequently incorporated into multi-herb formulations aimed at restoring ‘Qi’ and systemic vitality. From a pharmacological perspective, this traditional rationale aligns with emerging evidence that complex phytochemical mixtures may exert network-level modulation of neuroinflammatory and oxidative stress pathways. Such multi-target activity is particularly relevant in AD, where oxidative stress, mitochondrial dysfunction, neuroimmune activation, and amyloidogenic processing interact in a self-reinforcing pathological cascade.

Traditional Chinese Medicine (TCM) has emerged as a promising field for identifying bioactive phytochemicals that modulate multiple pathological pathways simultaneously. Within TCM, *Astragalus membranaceus* (also known as Huang Qi) is one of the most widely used tonic herbs, recognized for its immunomodulatory, antioxidant, and neuroprotective properties (Bourezzane et al., 2018; Q. Li et al., 2022; P. Liu et al., 2017). Its diverse array of phytochemicals – including saponins (such as Astragaloside IV (Costa et al., 2019), cycloestragenol cycloastragenol (Ikram et al., 2021)), flavonoids (e.g., formononetin (Tian et al., 2022) and kaempferol (Lekmine et al., 2021)), and polysaccharides (HUANG et al., 2013; X.-T. Li et al., 2012; Shi and Ma, 2024), has been shown to regulate signaling pathways relevant to neurodegeneration, particularly PI3K/Akt (Long et al., 2021), Nrf2/HO−1 (Bourezzane et al., 2018), and MAPK (Costa et al., 2019).

In recent years, multiple experimental studies have consistently demonstrated the potential of the compounds derived from *Astragalus spp.* in alleviating AD-related pathology. These phytochemicals exhibit antioxidative, anti-inflammatory, and anti-apoptotic properties; they also mitigate Aβ-induced neurotoxicity and enhance synaptic plasticity. Nonetheless, despite the growing number of studies, there remains a lack of consolidated understanding regarding their mechanistic profile and translational relevance. Furthermore, many investigations focus on *Astragalus spp.* as a component of complex TCM formulations, often diluting the attention. However, no prior systematic synthesis has evaluated the collective molecular mechanisms of compounds derived from *Astragalus spp.* in Alzheimer’s pathology.

The present systematic review aims at comprehensively summarizing and critically evaluating current literature on *Astragalus spp.* and its phytochemicals in the context of AD. By integrating evidence across molecular, cellular, and animal studies, this review seeks to:

1) Identify the primary *Astragalus* spp. phytochemicals investigated for anti-Alzheimer’s effects;.

2) Elucidate their reported mechanisms of neuroprotection and subsequent physiological outcomes; and.

3) Highlight knowledge gaps and future directions for translational and clinical research.

*Astragalus spp.* has a long-standing role in traditional medicine systems, particularly in East Asia, where it is used to promote vitality and support age-related health. From an ethnopharmacological perspective, such traditional usage patterns provide a valuable framework for identifying multi-target botanical interventions aligned with the complex pathophysiology of neurodegenerative disorders.

Although numerous experimental studies have investigated *Astragalus*-derived phytochemicals in Alzheimer's disease, the evidence remains dispersed across individual compounds and experimental models. Existing reviews have not comprehensively synthesized the convergent molecular mechanisms shared across *Astragalus*-derived phytochemicals and whole extracts in Alzheimer's disease. The present review addresses this gap by systematically integrating evidence from individual phytochemicals and whole extracts, identifying recurring molecular targets, signaling pathways, and biological processes that collectively contribute to neuroprotection. By emphasizing mechanistic convergence rather than individual compound-specific effects, this review provides an integrated framework that may guide future mechanistic investigations and translational research.

## 2 Methods

This systematic review conforms to PRISMA 2020 reporting guidelines.

### 2.1 Search strategy

Access limitations prevented screening subscription-based databases such as Web of Science and Scopus. Given feasibility constraints and the primary aim of mechanistic synthesis, PubMed and ScienceDirect were selected for initial screening and were later supplemented with extensive citation chain discovery via ResearchRabbit to increase coverage. Although database restriction represents a limitation, the search was complemented by extensive citation-network exploration to improve coverage of pharmacological and mechanistic studies.

A systematic search was conducted on October 17, 2025, across PubMed and ScienceDirect databases. The search query applied across both platforms was:

“(*Astragalus* [TITLE/ABSTRACT]) AND (Alzheimer’s [TITLE/ABSTRACT])”.

This initial search retrieved 135 records (96 from ScienceDirect and 39 from PubMed).

The completed PRISMA 2020 checklist is provided in the Supplementary Materials.

This review was not eligible for registration in PROSPERO because the database does not accept non-clinical or mechanistic systematic reviews. Therefore, preregistration was not performed.

### 2.2 Inclusion and exclusion criteria

Filters were applied to include only English-language articles with free full-text or open-access availability. After applying these filters, 42 articles remained (23 from ScienceDirect and 19 from PubMed).

Studies were included if they investigated the effects of *Astragalus spp.* or its phytochemicals in Alzheimer’s disease models (in vitro, in vivo, or clinical). Articles were excluded if they:

• Did not study phytochemicals that can be extracted from *Astragalus spp.* or
• Were confined to a certain geographical location as the scope of the study or
• Were systematic reviews or meta-analyses.

### 2.3 Screening and selection process

Duplicates were removed prior to screening. Abstracts were then reviewed for relevance, followed by full-text screening of eligible papers. Literature review papers and meta-analysis papers were excluded. This process yielded 10 studies (all from PubMed).

The remaining ScienceDirect records were excluded during screening because they did not satisfy the predefined eligibility criteria, represented duplicate publications already retrieved through PubMed, or were otherwise not relevant to the review objective.

Relevance was assessed according to the predefined PICOS eligibility criteria and the objective of identifying mechanistic evidence relating *Astragalus*-derived phytochemicals to Alzheimer's disease pathology.

The PICOS criteria for inclusion in the systematic review has been given in Table 1.

**Table 1:** PICOS criteria for inclusions in the systematic review. The PICOS framework outlines the predefined inclusion criteria based on population, intervention, comparator, outcomes, and study design for studies investigating Astragalus-derived phytochemicals in Alzheimer’s disease models.

**PICO Tool**	**Description**
**P**	AD models (in vitro, in vivo, limited clinical)
**I**	*Astragalus spp.* Phytochemicals/extracts
**C**	Untreated/vehicle controls
**O**	Anti-oxidative, anti-apoptotic, anti-inflammatory, neurotrophic, reduced τ and Aβ pathology, anti-cholinesterase, memory and cognitive improvement, iron homeostasis
**S**	Experimental studies (cell lines, animal models, pharmacokinetic papers)

### 2.4 Cross-referencing

To expand the dataset, cross-referencing was conducted using ResearchRabbit, which provided three discovery streams: ‘similar work’, ‘all citations’, and ‘all references’ for each included paper.

Within the ‘similar work’ stream, studies were screened until thematic saturation was achieved after ranking by relevance, while all records within citation and reference streams were screened to maximize retrieval of mechanistically related studies. In contrast, all results from the ‘all citations’ and ‘all references’ streams were thoroughly screened, as these sections tend to reveal directly related or foundational studies. Broader queries, being piloted, returned > 10⁴ irrelevant records; therefore, the narrowed strategy was adopted intentionally for specificity, supplemented by an intensive cross-referencing stage. This approach ensured a balance between breadth and depth, allowing the identification of both primary and secondary sources relevant to the neuroprotective and anti-Alzheimer’s roles of *Astragalus spp.* phytochemicals.

Cross-referencing identified an additional 20 studies, including 5 papers lacking the keywords “Alzheimer’s” and/or “*Astragalus*” in their abstracts – and 3 that did not mention “*Astragalus*” explicitly in the full text but were included due to their direct relevance to phytochemicals derived from *Astragalus spp.* and their anti-Alzheimer’s mechanisms. All cross-referenced articles were screened independently and were retained only if they met the same inclusion criteria as the original search. Cross-referenced articles were screened according to identical PICOS parameters. This ensured methodological consistency throughout the study selection process, irrespective of the source from which the articles were identified.

The final dataset represents the currently available mechanistic evidence meeting the predefined eligibility criteria and was considered appropriate for qualitative synthesis of convergent molecular pathways rather than quantitative meta-analysis.

In total, 30 studies were included in the final analysis, ranging from 2011 to 2024.

Although the final dataset comprised a relatively limited number of studies, this reflects both the current availability of mechanistic evidence and the predefined search strategy adopted for this review. To maximize coverage, database screening was supplemented through citation-network exploration using ResearchRabbit, including similarity-based discovery, citation tracking, and reference screening. The resulting dataset was considered sufficient for mechanistic synthesis because recurrent signaling pathways, phytochemical classes, and therapeutic outcomes were consistently reported across independent studies.

## 3 Results

### 3.1 Study selection

**Fig. 1:**
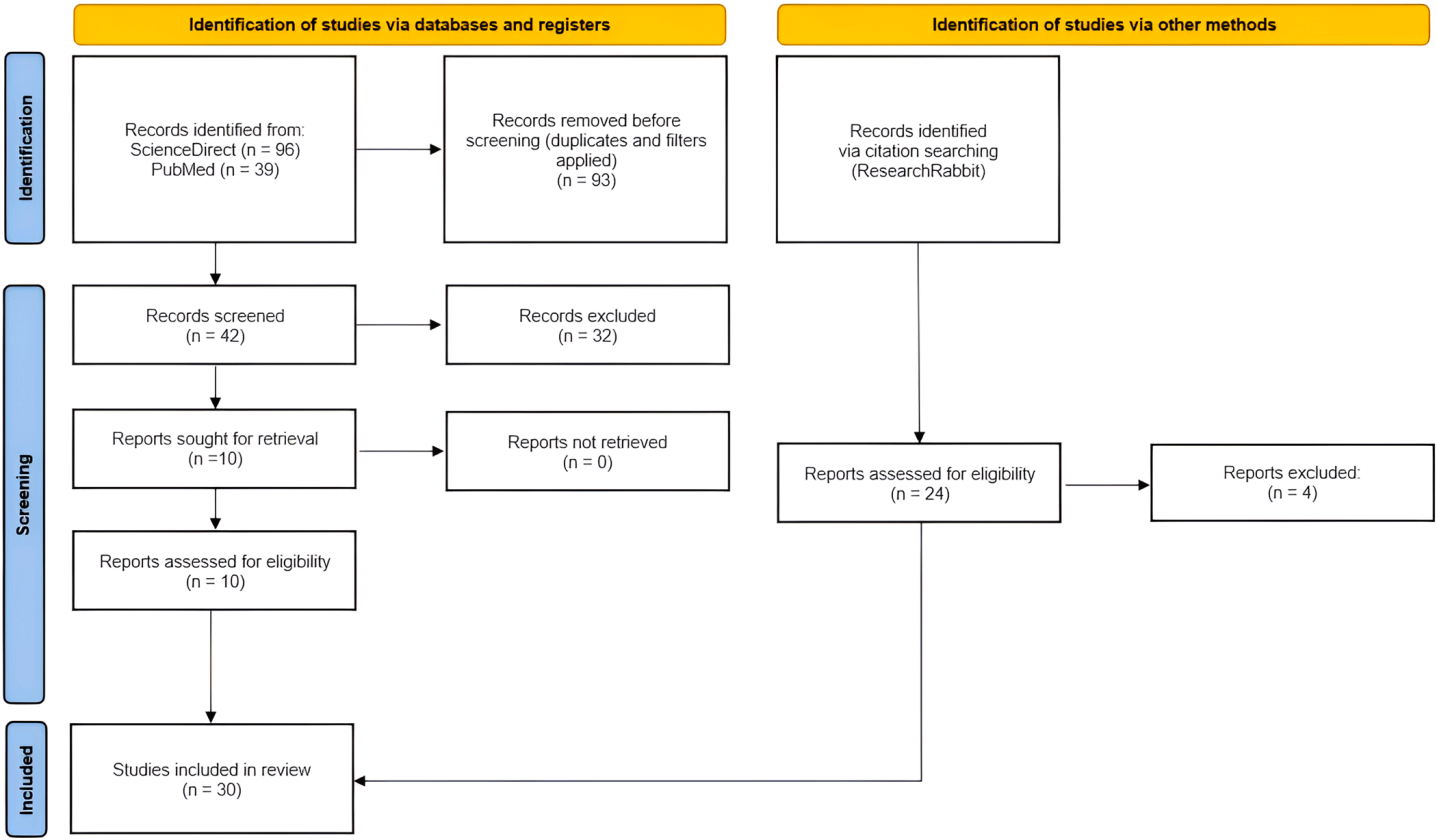
**PRISMA 2020 flow diagram of study selection.** Records were identified through database searching (PubMed and ScienceDirect) and citation searching (ResearchRabbit). Following screening and eligibility assessment, a total of 30 studies were included in the final systematic review.

A total of 30 studies were included in this review, spanning from 2011 to 2024. The study selection process is summarized in the PRISMA 2020 flow diagram (Fig. 1).

### 3.2 Study characteristics

The included studies encompassed a range of experimental designs, including in vitro, in vivo, and a limited number of clinical investigations. Most studies focused on cellular and animal models of AD, particularly those simulating β-amyloid (Aβ) toxicity or oxidative stress-induced neuronal injury.

**Fig. 2:**
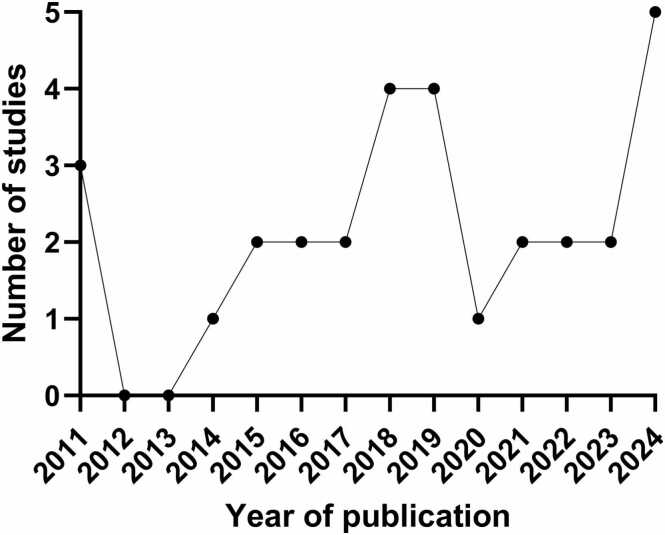
Temporal distribution of included studies (2011–2024)**.** The number of studies published per year demonstrates trends in research activity over time.

**Fig. 3:**
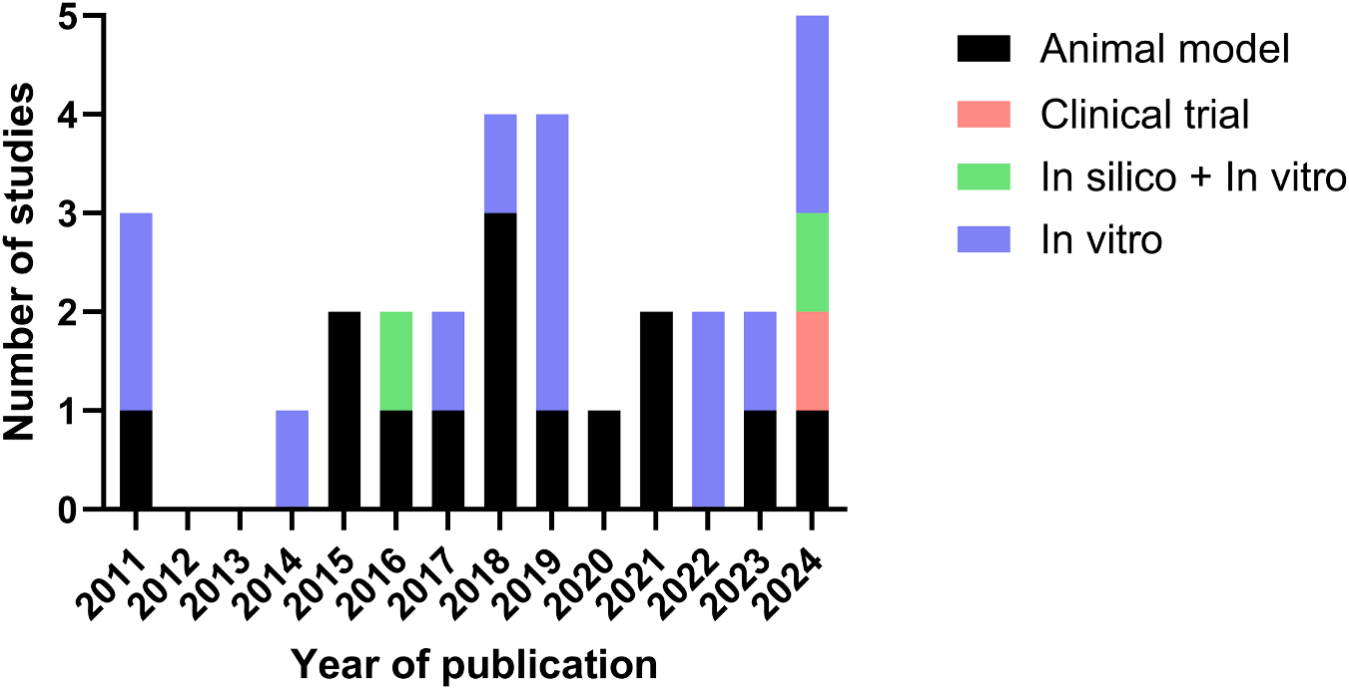
Temporal distribution of study types in included studies (2011–2024). The number of studies by experimental design (in vitro, in vivo/animal models, combined in silico–in vitro approaches, and clinical trials) is shown across the study period.

**Fig. 4:**
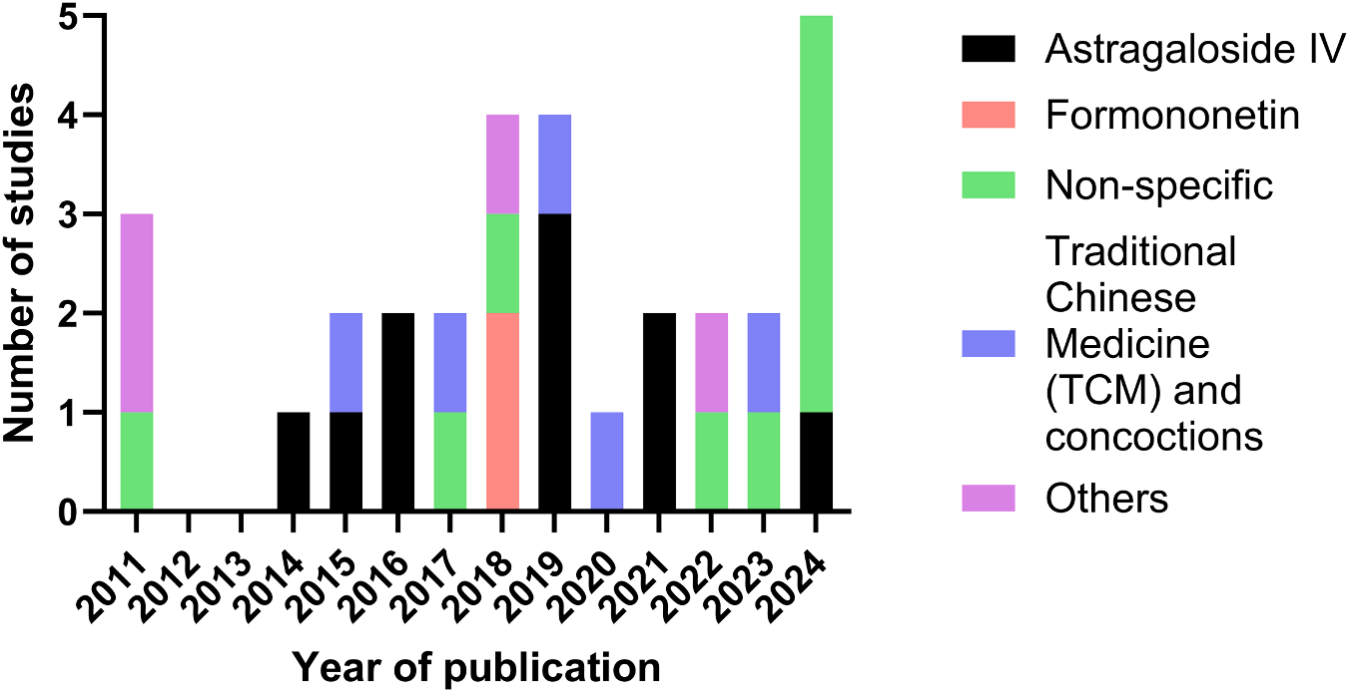
Temporal distribution of phytochemical-focused studies (2011–2024)**.** The frequency of studies investigating key *Astragalus*-derived phytochemicals over time is shown. Less frequently studied compounds (caffeic acid, coumestrol, and kaempferol) were grouped under “Others” for clarity.

A year-wise analysis revealed a noticeable rise in publications post 2017, with an upward trend from 2022 onwards, marking the growing global recognition of multi-target natural therapeutics in neurodegeneration research (Fig. 2). Moreover, clinical trials as a study type in the niche seem to have begun – suggesting increasing translational interest (Fig. 3). A decreased focus on individual phytochemicals can be noticed with the rise in number of studies revolving around TCM concoctions and non-specific extract mixtures (Fig. 4).

**Fig. 5:**
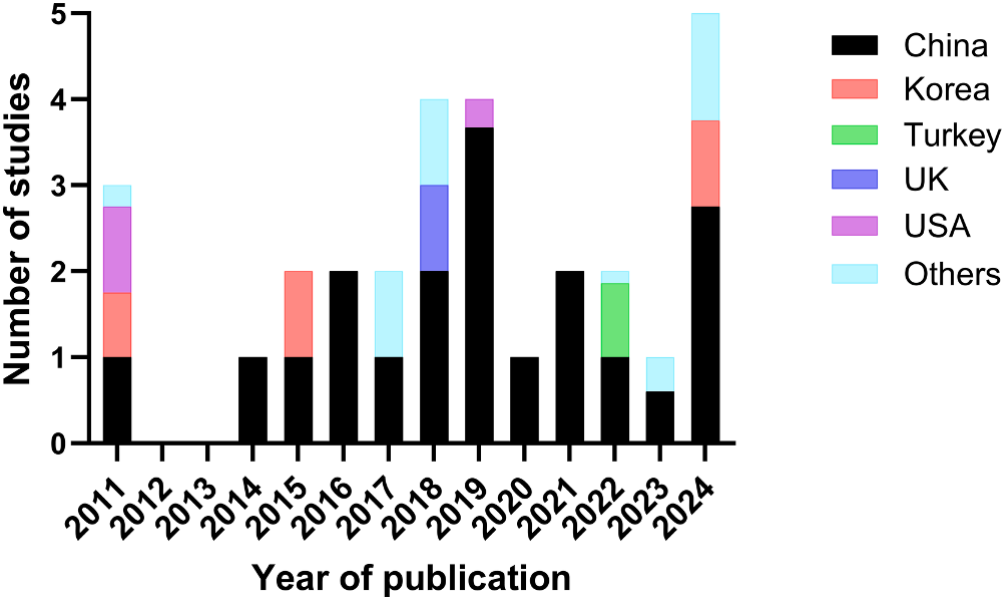
Temporal and geographical distribution of included studies (2011–2024)**.** Countries with low publication frequency (≤1 study) were grouped under “Others” for visualization clarity. These include Algeria, Canada, India, Italy, Nepal, Saudi Arabia, Spain, Taiwan, and Tunisia.

**Fig. 6:**
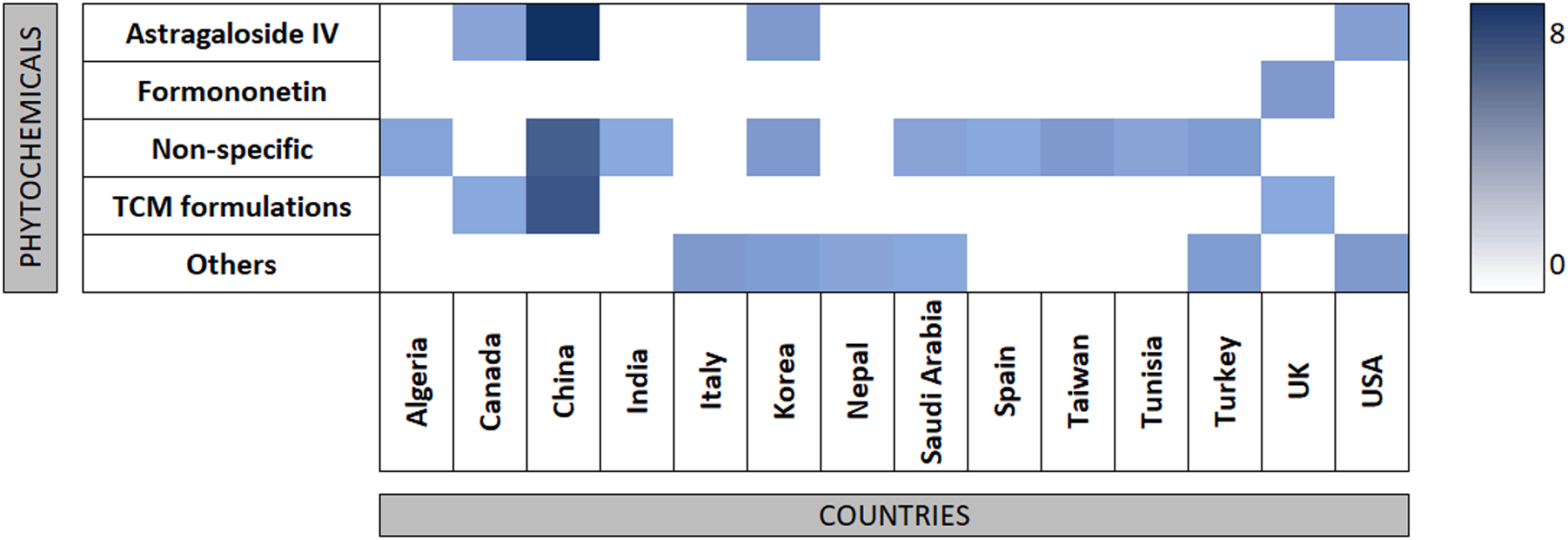
Geographical distribution of phytochemical-focused studies in Alzheimer’s disease**.** Heatmap showing the distribution of studies investigating major *Astragalus*-derived phytochemicals across countries. Color intensity corresponds to the number of studies, with darker shades indicating higher publication frequency. Less frequently studied compounds (caffeic acid, coumestrol, and kaempferol) were grouped under “Others” for clarity.

Geographically, the majority of publications originated from China, followed by contributions from South Korea, Japan, and several Western countries, underscoring the herb’s traditional prominence in East Asian medicine. Interestingly, more countries are participating in the research on the medicinal herb genus; indicating broader global research engagement supporting wider translational relevance of *Astragalus spp.* in various geographical contexts (Fig. 5). Lastly, it can be seen that non-specific phytochemical compositions, TCM, and Astragaloside IV are the major areas being explored by countries who have recently begun to investigate *Astragalus spp.* in the context of AD (Fig. 6).

### 3.3 Synthesized findings

Detailed study-level characteristics are provided in Supplementary Table S1

#### 3.3.1 *Astragalus* phytochemicals (n = 25)

Among the 30 included studies, 25 investigated isolated or semi-purified phytochemicals derived from *Astragalus spp*. These studies primarily explored the molecular mechanisms underlying neuroprotection, targeting oxidative stress, mitochondrial dysfunction, neuroinflammation, and synaptic integrity. Compounds such as astragaloside IV and formononetin were the most extensively studied, demonstrating consistent effects across multiple AD-related signaling pathways.

##### 3.3.1.1 Astragaloside IV (AS-IV) (n = 10)

AS-IV emerged as the most frequently studied bioactive component, investigated across 10 studies. It consistently demonstrated antioxidative, anti-apoptotic, and autophagy-regulating properties. Mechanistically, Astragaloside IV was shown to modulate the PI3K/Akt (Liu et al., 2016), Nrf2/HO−1 (Chen et al., 2021, Liu et al., 2016; J. Yu et al., 2019), MAPK (Kim et al., 2015, Ma and Xiong, 2019, Wang et al., 2021; J. Yu et al., 2019) contributing to reduced oxidative stress, inhibition of neuronal apoptosis, and enhanced mitochondrial stability.

Experimental models primarily included Aβ-induced neurotoxicity in amnesia-like rat models (Yanfang et al., 2024), SK-N-SH cells (Sun et al., 2014), and APP/PS1 transgenic mice (W. Yu et al., 2019), all of which demonstrated significant improvements in memory performance. An additional study reported that AS-IV modulated the Notch signaling pathway, thereby conferring neurotrophic properties and promoting neural stem cell proliferation and differentiation (Haiyan et al., 2016).

Overall, despite variations in experimental models, AS-IV consistently converges on a core set of mechanisms involving oxidative-stress reduction, microglial modulation, mitochondrial stabilization, and activation of prosurvival signaling pathways such as PI3K/Akt and Nrf2/HO−1. This consistency across models suggests that AS-IV acts as a multi-target neuroprotective agent rather than exerting a single pharmacological effect.

**Fig. 7:**
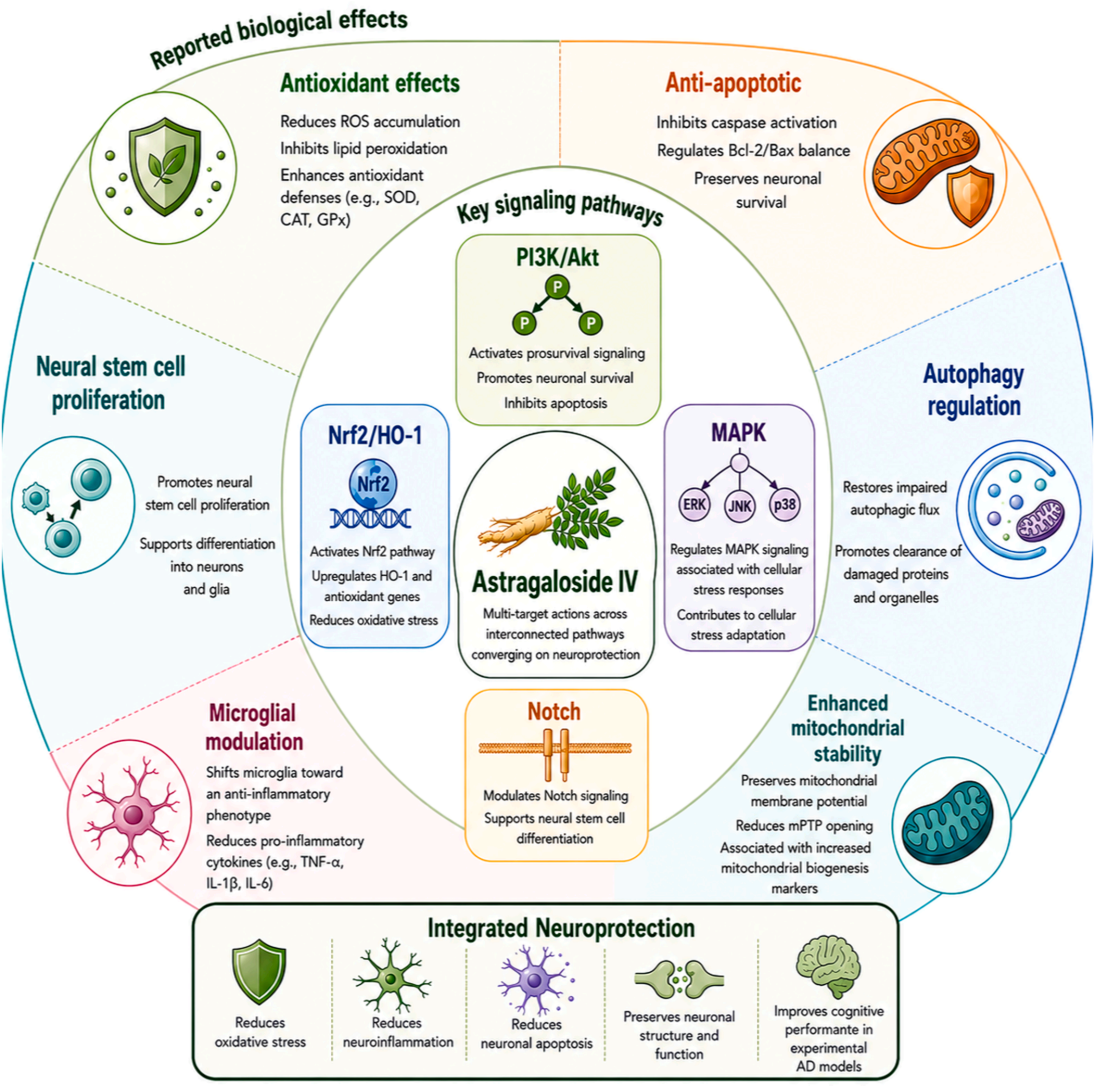
Mechanistic convergence underlying the neuroprotective actions of Astragaloside IV**.** Astragaloside IV exerts multi-target neuroprotective effects by modulating key signaling pathways and cellular processes, including PI3K/Akt, Nrf2/HO−1, MAPK, and Notch signaling. These interconnected mechanisms collectively reduce oxidative stress, neuronal apoptosis, and mitochondrial dysfunction while promoting autophagy, neural stem cell proliferation, and neuronal survival, microglial modulation, thus ultimately contributing to neuroprotection in experimental models of Alzheimer's disease.

A schematic summary of these convergent molecular mechanisms is presented in Fig. 7.

##### 3.3.1.2 Formononetin (FMN) (n = 2)

FMN, a naturally occurring isoflavonoid, has emerged as a promising anti-Alzheimer’s candidate due to its multifaceted mechanisms targeting both amyloid pathology and neuroinflammation. In APP/PS1 transgenic mice, FMN significantly improved learning and memory performance by suppressing β-amyloid (Aβ) production, attenuating RAGE-dependent inflammatory signaling, and enhancing LRP1-mediated Aβ clearance across the blood–brain barrier (BBB) (Fei et al., 2018).

An in vitro study demonstrates that FMN effectively inhibits neuroinflammatory cascades in LPS-stimulated BV2 microglia by downregulating proinflammatory mediators (TNF-α, IL-1β, IL-6, iNOS, and COX−2) through ERβ-dependent suppression of the NF-κB signaling pathway, thus protecting neurons from microglia-induced toxicity (El-Bakoush & Olajide, 2018).

Collectively, these findings highlight formononetin’s strong therapeutic promise for AD by simultaneously targeting amyloid metabolism, neuroinflammation, and BBB function.

Together, these findings indicate that formononetin exerts convergent neuroprotection through simultaneous modulation of amyloid metabolism, microglial-driven inflammation, and BBB-associated transport systems. Its actions consistently intersect with estrogen receptor signaling and NF-κB suppression, forming a coherent mechanistic profile across diverse models.

**Fig. 8:**
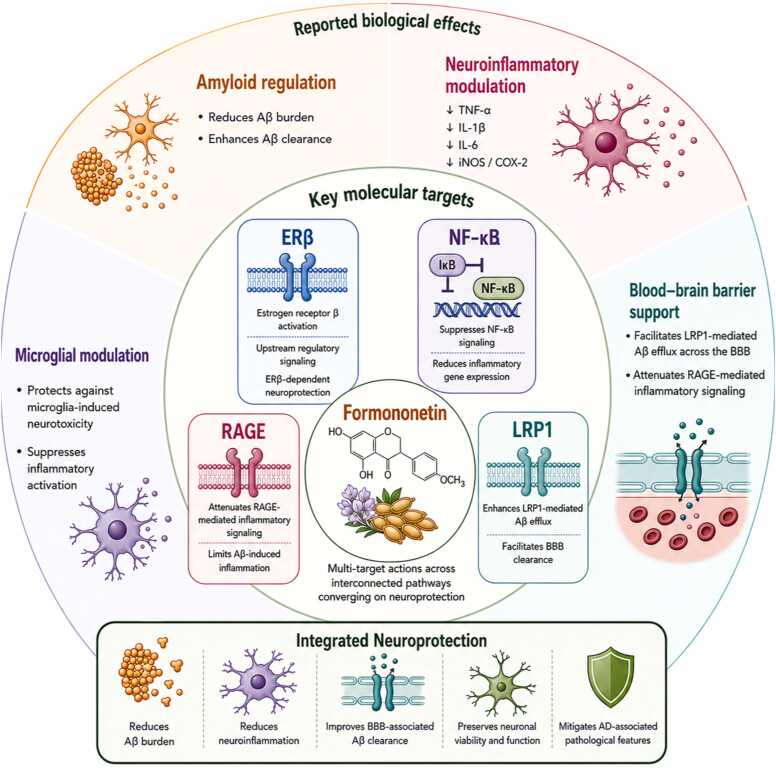
Mechanistic convergence underlying the neuroprotective actions of Formononetin**.** Formononetin exerts coordinated neuroprotective effects through modulation of estrogen receptor β (ERβ), NF-κB, RAGE, and LRP1-mediated signaling. Together, these molecular targets regulate amyloid metabolism, neuroinflammatory responses, blood-brain barrier-associated Aβ clearance, and microglial activation, forming a coherent mechanistic profile that contributes to neuroprotection in experimental models of Alzheimer's disease.

These interconnected mechanisms are summarized schematically in Fig. 8.

##### 3.3.1.3 Minor phytochemicals (n = 4)

In addition to well-studied phytochemicals, several minor botanical compounds have shown promising neuroprotective effects against AD through diverse mechanisms.

While studied less extensively, these minor phytochemicals converge on pathways regulating oxidative stress and inflammation. Collectively, they activate Nrf2/HO−1 signaling, suppress NF-κB-mediated neuroinflammation, and modulate apoptotic and cholinergic systems – revealing a shared antioxidant and anti-inflammatory foundation across structurally distinct compounds.

###### 3.3.1.3.1 Caffeic acid

Caffeic acid phenethyl ester (CAPE), a polyphenol found in honeybee propolis as well as *Astragalus* plants, mitigates Aβ1–42-induced neurotoxicity by activating the Nrf2/HO−1 antioxidant pathway and modulating glycogen synthase kinase 3β, thereby reducing oxidative stress, neuroinflammation, and cognitive decline in mice (Morroni et al., 2018).

###### 3.3.1.3.2 Coumestrol

Coumestrol, a coumestan phytoestrogen, exhibits potent antioxidant and anticholinergic activities, with significant inhibition of acetylcholinesterase and butyrylcholinesterase – enzymes implicated in AD pathology – suggesting its therapeutic relevance (Durmaz et al., 2022).

###### 3.3.1.3.3 Kaempferol

Flavonoids such as kaempferol, though weak direct inhibitors of β-secretase (BACE−1), downregulate its expression via NFκB inhibition, thereby reducing Aβ production. Kaempferol exerts anti-inflammatory effects by suppressing TLR4-mediated NFκB and MAPK signaling, lowering pro-inflammatory cytokines and ROS levels (Paris et al., 2011, Park et al., 2011). Collectively, these minor phytochemicals offer multifaceted neuroprotective strategies, highlighting their potential as adjunctive agents in AD management.

Although mechanistically diverse, these minor compounds share a unifying pattern of modulating oxidative stress, inflammatory signaling, and apoptotic pathways. Their convergence on Nrf2/HO−1 activation and NF-κB suppression mirrors the broader mechanistic themes observed across the *Astragalus spp.* phytochemical spectrum.

**Fig. 9:**
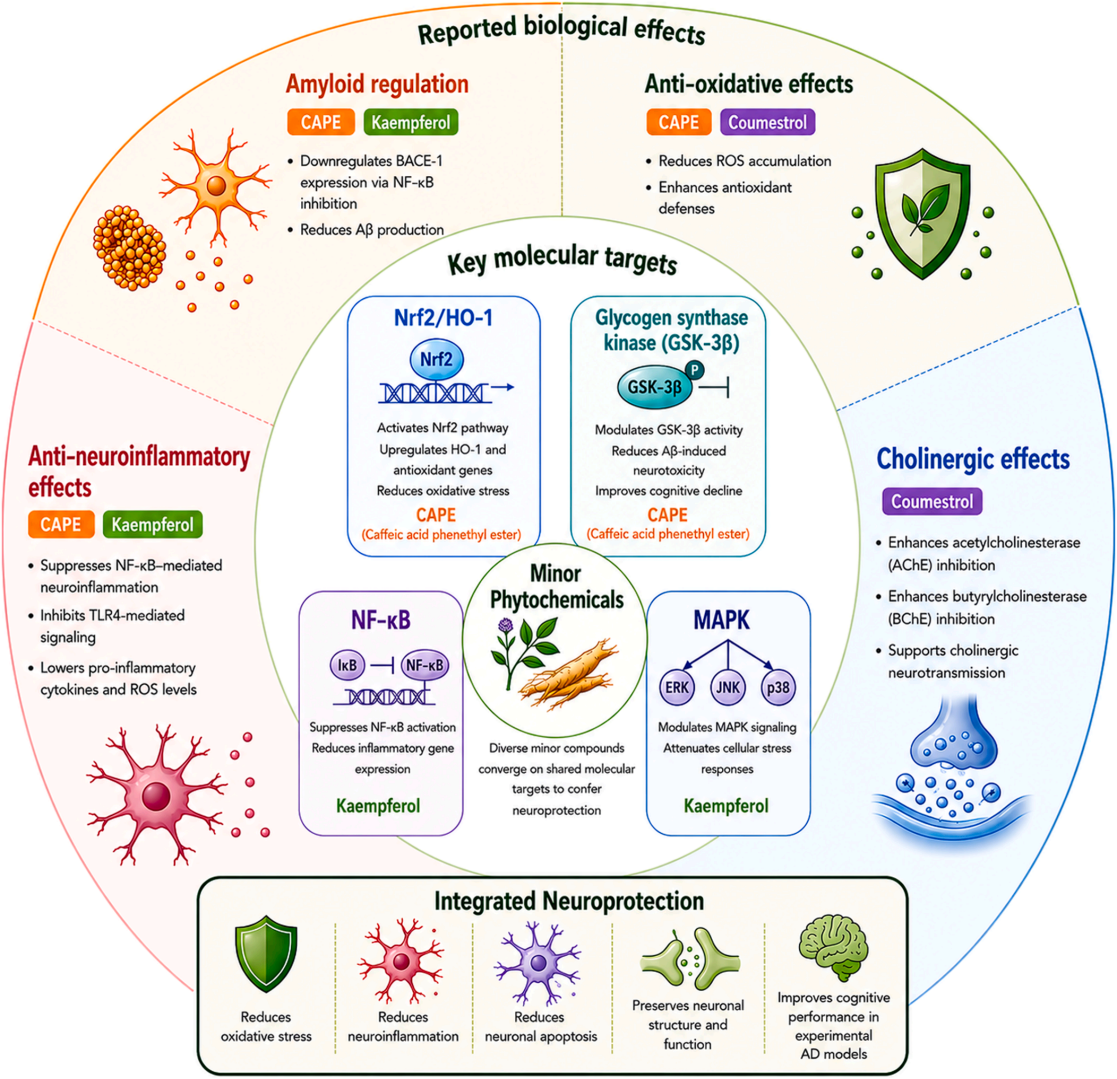
Mechanistic convergence among selected minor *Astragalus*-derived phytochemicals**.** Although less extensively investigated, minor phytochemicals – including caffeic acid phenethyl ester (CAPE), coumestrol, and kaempferol – converge on shared molecular mechanisms involving Nrf2/HO−1 activation, glycogen synthase kinase−3β (GSK−3β) modulation, NF-κB suppression, and MAPK regulation. Despite their mechanistic diversity, these compounds collectively promote antioxidant, anti-inflammatory, cholinergic, and amyloid-modulating effects that converge on integrated neuroprotection in Alzheimer's disease.

A schematic overview of these shared molecular targets and biological effects is presented in Fig. 9.

##### 3.3.1.4 Holistic/unspecified extracts (n = 9)

A growing body of evidence suggests that *Astragalus spp.* and their bioactive derivatives exert broad neuroprotective and anti-Alzheimer’s effects through multi-target mechanisms encompassing antioxidant, anti-inflammatory, anti-apoptotic, and metabolic pathways. *Astragalus spp.* extracts and polysaccharides have been shown to counter stress-induced neuronal apoptosis by reducing caspase−3 and cytochrome c expression, thereby preserving hippocampal neurons and improving learning and memory in animal models (W.-Z. Li et al., 2011). *Astragalus membranaceus* polysaccharides further alleviate metabolic stress-induced insulin resistance, neuroinflammation, and gliosis in transgenic AD mice, reflecting its potential in sporadic, metabolically driven AD (Huang et al., 2017). Co-administration of *Astragalus spp.* with icariin and puerarin reduced cortical iron overload, oxidative damage, and inflammatory cytokine levels, emphasizing its antioxidant synergy (Y. Zhang et al., 2018). Extracts of various *Astragalus spp.* exhibit strong acetylcholinesterase and butyrylcholinesterase inhibition, along with rich phenolic and flavonoid content, suggesting cholinergic system protection and antioxidant potency (Güven et al., 2023, Lekmine et al., 2024). Systems-pharmacology approaches revealed that *HuangQi* (*Astragalus membranaceus*) acts on multiple AD-related targets, including TNF, AGE-RAGE, and NF-κB signaling, while protecting neurons from Aβ₁₋₄₂-induced cytotoxicity (Lv et al., 2024). *Astragalus spp.* extract also modulates matrix metalloproteinases, preserving synaptic integrity and reducing neurodegenerative progression (Z. Li et al., 2022). Moreover, roasting of *Astragalus spp.* enhances its neuroprotective profile by activating the AKT/CREB/BDNF pathway, boosting antioxidant enzymes, and reducing Aβ-induced ROS and apoptosis (Cheng et al., 2024). Finally, a recent pragmatic clinical trial aims to evaluate the safety and efficacy of add-on *Astragalus membranaceus* therapy in AD patients with orthostatic hypotension, potentially extending its use from preclinical promise to clinical validation (Ji et al., 2024).

Whole extracts and polysaccharides of *Astragalus spp.* demonstrate multi-pathway neuroprotection, integrating antioxidant, anti-apoptotic, and metabolic regulation. Their broad activity on Nrf2, PI3K/Akt, and CREB/BDNF signaling highlights synergistic effects likely derived from the herb’s phytochemical diversity, supporting its traditional use as a tonic in cognitive disorders.

Across these studies, the multifaceted nature of whole *Astragalus spp.* extracts yields a recurring mechanistic signature involving antioxidant defense, mitochondrial protection, and neuroimmune regulation. The breadth of active constituents likely contributes to network-level pharmacological effects not attributable to any single compound.

**Fig. 10:**
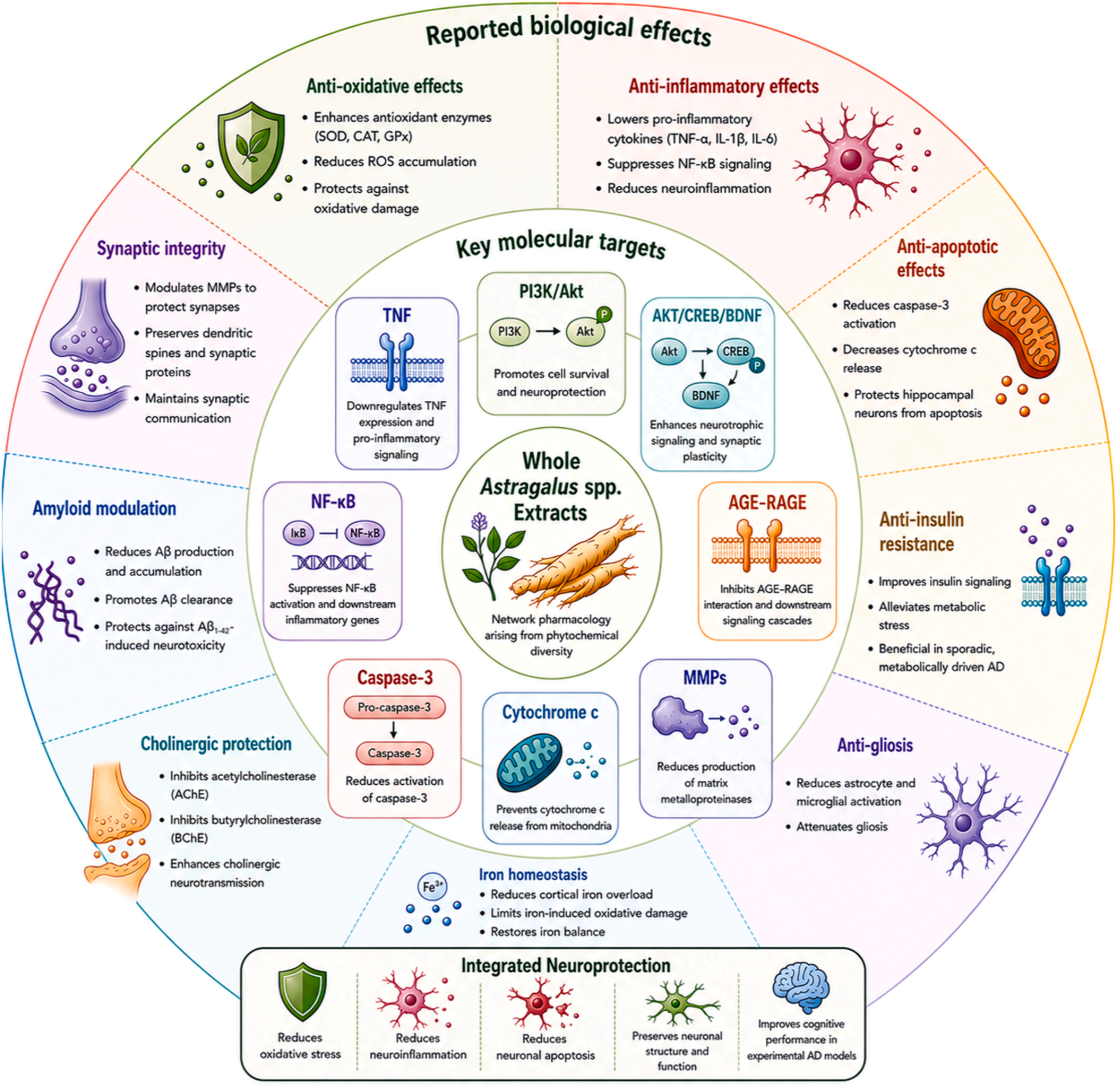
Mechanistic convergence underlying the neuroprotective actions of whole *Astragalus* spp. extracts and polysaccharides**.** Whole *Astragalus* spp. extracts and polysaccharides exhibit broad neuroprotective activity through simultaneous modulation of multiple molecular targets, including PI3K/Akt, AKT/CREB/BDNF, AGE-RAGE signaling, matrix metalloproteinases (MMPs), cytochrome c, caspase−3, NF-κB, and TNF. These network-level interactions collectively regulate oxidative stress, inflammation, apoptosis, insulin resistance, gliosis, iron homeostasis, cholinergic function, amyloid pathology, and synaptic integrity, illustrating the systems-level pharmacology arising from the phytochemical diversity of whole *Astragalus* preparations.

The resulting systems-level pharmacological network is summarized in Fig. 10.

#### 3.3.2 Traditional Chinese medicine and concoctions (n = 5)

TCM formulations and multi-herb decoctions have shown considerable promise in counteracting AD pathology through multi-target, synergistic mechanisms that integrate antioxidant, anti-inflammatory, and neurotrophic actions. It is known that one of the hallmark characteristics of AD is the disruption in iron homeostasis (Ghaderi et al., 2024). Combined extracts of *Epimedium*, *Astragalus* (species not specified), and *Radix Puerariae* improved cognition, reduced β-amyloid (Aβ) plaque burden, and restored brain iron homeostasis in APP/PS1 transgenic mice by regulating divalent metal transporter 1 (DMT1) and ferroportin 1 (FPN1) expression, thereby mitigating iron overload – a key factor in AD neurotoxicity (Dong et al., 2015). The classic herbal decoction Danggui Buxue Tang (DBT), composed of *Astragali* Radix and *Angelicae sinensis* Radix, demonstrated neurotrophic and neuroprotective potential through upregulation of BDNF, NGF, and GDNF, activation of the MAPK/ERK-CREB pathway, and suppression of Aβ-induced apoptotic markers such as cleaved-caspase−3 and PARP, suggesting its role in reducing neuronal apoptosis and enhancing neuroplasticity (A. G. W. Gong et al., 2017; G. Gong et al., 2019). Similarly, LongShengZhi Capsule (LSZ), approved by the Chinese FDA, ameliorated Aβ and p-Tau accumulation, inhibited β- and γ-secretase activity, and modulated NF-κB-mediated inflammation, while promoting antioxidant enzyme activity, indicating its efficacy in both oxidative and amyloidogenic pathways (Yin et al., 2020). Lastly, Jia-Wei-Kai-Xin-San (JWKXS) improved memory and neuronal morphology in MCI (mild cognitive impairment) SAMP8 mouse models by suppressing TLR4/NF-κB signaling, inhibiting NLRP3 inflammasome activation, and blocking intrinsic/extrinsic apoptotic pathways, collectively reducing neuroinflammation and apoptosis (X. Zhang et al., 2023). Altogether, these findings illustrate how TCM concoctions operate in a network-pharmacology manner, targeting interconnected molecular pathways that underlie AD – offering a holistic and integrative framework for future anti-Alzheimer’s therapeutics.

The multi-herb formulations consistently demonstrated network-wide modulation of neurotrophic factors, inflammatory cascades, and amyloid-processing pathways. *Astragalus spp.* frequently appears as a central contributor within these formulations, reinforcing its role as a key hub compound driving synergistic neuroprotective effects.

Across heterogeneous experimental models, a consistent convergence was observed on oxidative stress regulation, neuroinflammatory modulation, and mitochondrial preservation.

### 3.4 Phytochemical heterogeneity and standardization challenges

A key limitation across the included studies is the substantial heterogeneity in *Astragalus* spp. phytochemical composition and extract standardization. Although Astragaloside IV and formononetin were frequently investigated as isolated compounds, a considerable proportion of studies relied on holistic extracts, polysaccharide fractions, or multi-component preparations without consistent reporting of extraction procedures, purity, or quantitative phytochemical profiling. This variability complicates cross-study comparison and limits mechanistic attribution to specific compounds.

Batch-to-batch variability is a particularly relevant concern in ethnopharmacological research, as *Astragalus* phytochemical content is influenced by species identity, cultivation conditions, harvesting season, plant part used, and post-harvest processing methods. Such factors may alter the relative abundance of saponins, flavonoids, and polysaccharides, thereby affecting biological outcomes in experimental models.

Moreover, several studies did not report the use of marker compounds or analytical validation (e.g., LC-MS/MS, HPLC, or UPLC-based profiling) to confirm phytochemical composition. In the absence of standardized quality control, it remains difficult to determine whether observed neuroprotective effects reflect reproducible pharmacological activity or preparation-specific characteristics. This is especially relevant for studies evaluating “unspecified extracts” or Traditional Chinese Medicine formulations, where synergistic effects may occur but individual contributions remain unclear.

Overall, the lack of standardized phytochemical characterization represents a major translational bottleneck. Future studies should incorporate rigorous phytochemical validation, including quantification of marker constituents (e.g., Astragaloside IV, cycloastragenol, formononetin), purity reporting, and reproducible extraction protocols, to strengthen mechanistic interpretation and improve comparability across experimental systems.

### 3.5 Risk of bias

A narrative risk-of-bias evaluation was performed because the included studies comprised heterogeneous study models and approaches (in vitro models, animal studies, and phytochemical characterization papers), thus rendering formal scoring tools unsuitable. Instead, common methodological limitations were summarized across study types, emphasizing transparency and reproducibility.

A narrative risk-of-bias summary is provided in Table 2.

**Table 2:** Narrative assessment of risk of bias across different study types included in the review. Summary of common methodological limitations and overall risk of bias associated with in vitro, in vivo, phytochemical, and in silico studies investigating Astragalus-derived compounds in Alzheimer’s disease.

**Study type**	**Common concerns**	**Bias risk**	Reason
**In vitro studies**	Lack of blinding; selective reporting; no dose-response justification	Low-Moderate	Methods clearly describe treatment conditions, but blinding is typically absent in cell assays.
**Animal studies**	Randomization rarely reported; small sample sizes; missing blinding	Moderate	Most studies report clear behavioral/biochemical outcomes but omit allocation concealment or blinding.
**Phytochemical isolation/characterization papers**	Purity reporting varies; batch variability	Low-Moderate	Chemical procedures are well described, but purity QC isn’t standardized across studies.
**Mechanistic network or molecular docking studies**	In silico biases (database selection, scoring functions)	Low	Computational choices introduce bias, but methods are transparent and reproducible.

**Fig. 11:**
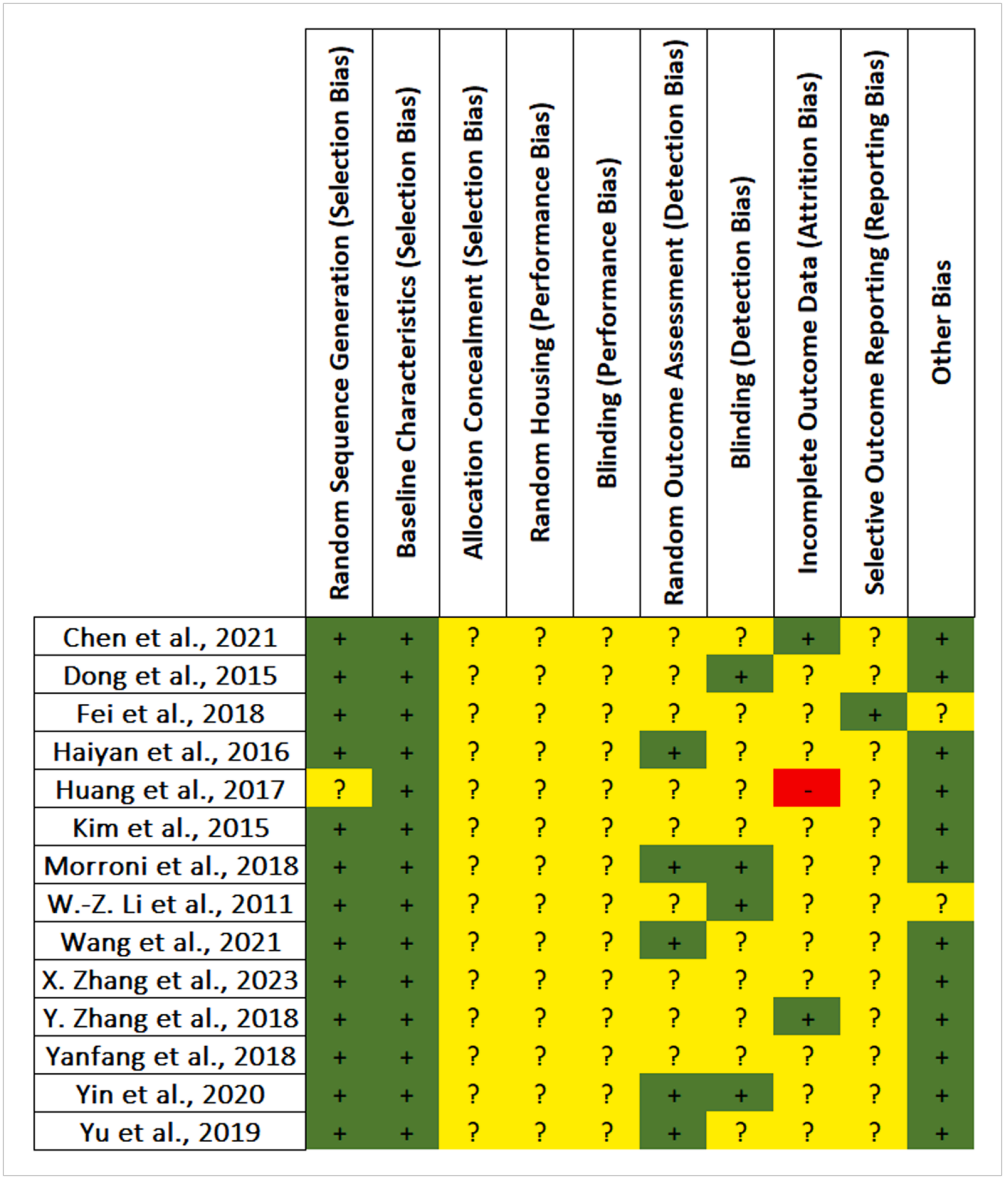
SYRCLE risk of bias summary for included animal studies**.** Risk of bias summary for included animal studies based on the SYRCLE tool. Each domain was assessed as low, high, or unclear risk of bias. The figure presents the proportion of studies meeting each criterion across domains including selection bias, performance bias, detection bias, attrition bias, reporting bias, and other sources of bias.

For animal studies, a SYRCLE-based risk of bias assessment is additionally provided in the form of Fig. 11.

### 3.6 Mechanistic summary

**Fig. 12:**
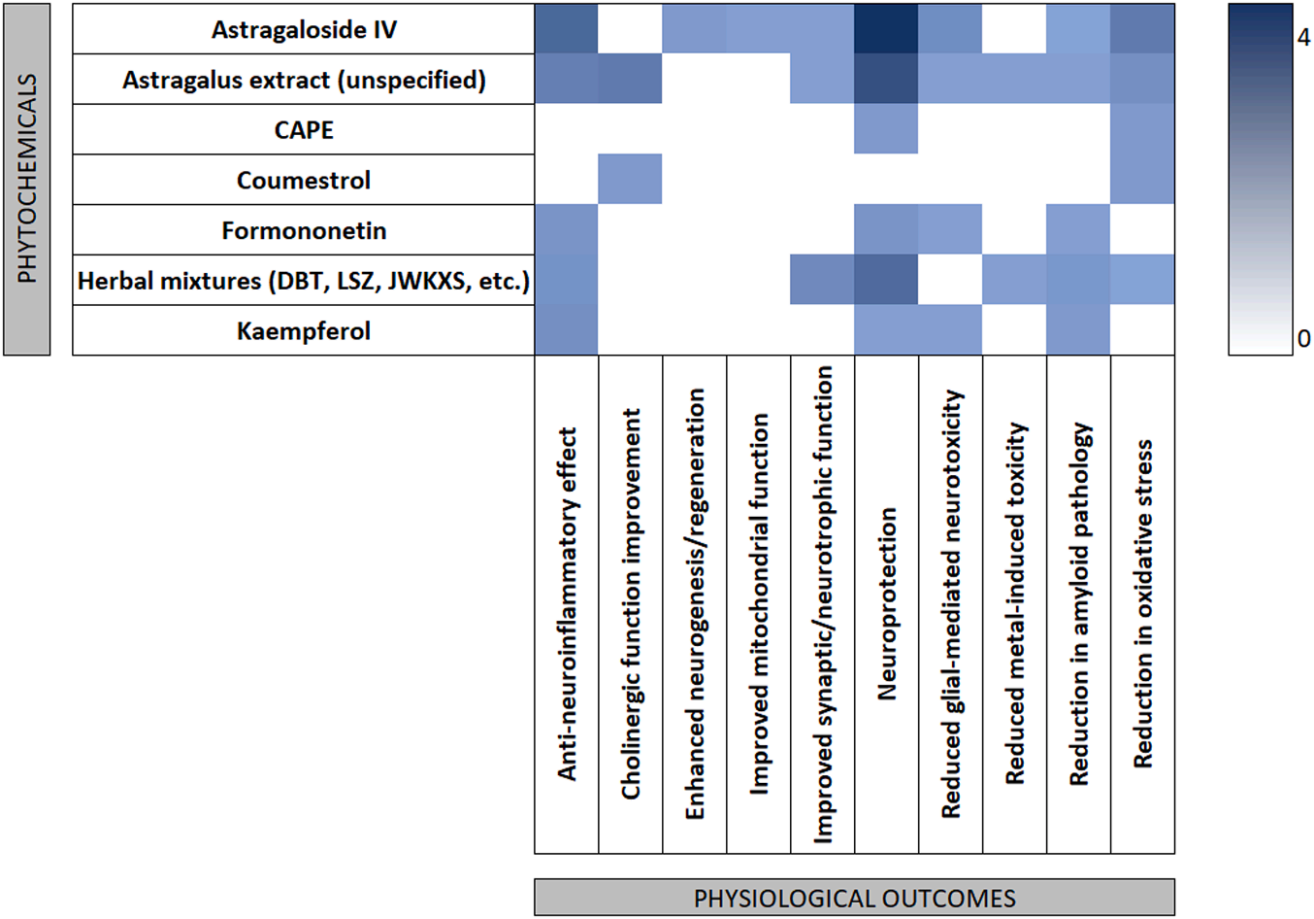
Summary of reported physiological effects of *Astragalus*-derived phytochemicals in Alzheimer’s disease**.** Heatmap summarizing the reported physiological effects of *Astragalus*-derived phytochemicals across key outcome domains relevant to Alzheimer’s disease. Color intensity reflects the number of studies supporting each effect. Multi-component formulations and herbal mixtures are included where applicable. The clinical trial by Cheng et al. (2024) was not included in this heatmap, as it did not report specific physiological outcome measures aligned with the categories presented.

The physiological outcomes of the various phytochemical (classes) have been summarized in Fig. 12.

A summary of the modes of action mentioned in the studies analyzed and the physiological outcomes they confer is given in Table 3.

**Table 3:** Summary of therapeutic outcome domains and associated molecular mechanisms of Astragalus-derived phytochemicals in Alzheimer’s disease. The table integrates reported therapeutic effects with their underlying molecular mechanisms across in vitro and in vivo Alzheimer’s disease models.

**Therapeutic Outcomes**	**Related Mechanisms**
**Anti-oxidative**	Decreased ROS generation Increased Nrf2/HO−1 activation Increased antioxidant enzyme activity (SOD, GSH-Px, CAT) Decreased mitochondrial oxidative stress Decreased MAPK-driven oxidative injury
**Anti-apoptotic**	Decreased caspase−3 and cytochrome c Increased PI3K/Akt survival signaling Decreased JNK/p38-mediated apoptosis Decreased PARP cleavage Increased CREB/BDNF pro-survival signaling
**Anti-inflammatory**	Decreased NF-κB activation Decreased NLRP3 inflammasome activity Decreased TLR4 signaling Decreased microglial M1 activation Increased M2 microglial polarization Decreased NADPH oxidase expression Decreased pro-inflammatory cytokines (TNF-α, IL-6)
**Neurotrophic**	Increased BDNF expression Increased NGF and GDNF Increased TrkB–CREB signaling Increased ERK/CREB neurotrophic activation
**Reduced τ and Aβ pathology**	Decreased APP processing toward Aβ Decreased BACE−1 expression Increased LRP1-mediated Aβ clearance Decreased RAGE-mediated Aβ influx/inflammation Decreased β- and γ-secretase activity
**Anti-cholinesterase**	Decreased acetylcholinesterase (AChE) activity Decreased butyrylcholinesterase (BChE) activity
**Iron homeostasis**	Decreased DMT1 (iron import) Increased FPN1 (iron export) Decreased iron-overload–induced oxidative stress

A detailed summary of included studies, showing model types, molecular mechanisms and therapeutic outcomes, is provided in Supplementary Table S1.

## 4 Discussion

From a phytotherapeutic perspective, the findings of this review highlight the potential of *Astragalus*-derived compounds as multi-target modulators of neurodegenerative processes. Mechanistic convergence across studies recurringly highlights modulation of oxidative stress, neuroinflammation, and mitochondrial dysfunction. Compounds such as Astragaloside IV and Formononetin consistently activated PI3K/Akt, Nrf2/HO−1, and MAPK pathways – signaling cascades integral to neuronal survival and synaptic maintenance. These overlapping mechanisms suggest compounds derived from *Astragalus spp.* act through multi-target synergy rather than single-pathway modulation, thus aligning with the systems-pharmacology perspective often attributed to TCM. Despite promising preclinical evidence, the translation of these findings into clinical practice remains limited, highlighting the need for well-designed human studies evaluating standardized *Astragalus* preparations.

### 4.1 Mechanistic convergence and network pharmacology relevance

This systematic review consolidates evidence that *Astragalus* spp.-derived phytochemicals exert multi-target neuroprotective actions in Alzheimer’s disease (AD) models. Across diverse experimental systems, mechanistic convergence was repeatedly observed around oxidative stress regulation, neuroimmune modulation, and mitochondrial preservation. Notably, Astragaloside IV and formononetin consistently intersected with key signaling pathways such as PI3K/Akt, Nrf2/HO−1, and MAPK cascades, which collectively govern neuronal survival, inflammatory signaling, and antioxidant defense.

The recurring involvement of these pathways suggests that *Astragalus* spp. phytochemicals may act through network-level modulation rather than isolated single-target mechanisms. This aligns with contemporary pharmacological understanding of complex plant-derived compounds, where pleiotropic molecular effects may provide therapeutic advantage in multifactorial disorders such as AD. In particular, the ability to simultaneously attenuate oxidative damage, suppress pro-inflammatory mediators, and reduce apoptotic signaling reflects a mechanistic profile that is pharmacologically coherent with the systems-based rationale underlying Traditional Chinese Medicine (TCM).

Furthermore, studies investigating multi-herb formulations containing *Astragalus* spp. provide additional support for the network pharmacology paradigm, as these preparations demonstrated combined effects on amyloid processing, neurotrophic signaling, and inflammatory regulation. Such findings reinforce the hypothesis that *Astragalus* spp. may serve as a key hub herb contributing to broader neuroprotective synergy within TCM formulations.

### 4.2 Translational barriers: bioavailability, BBB penetration, and dosing variability

Despite encouraging mechanistic consistency across preclinical models, several translational barriers limit clinical inference. A major concern is the uncertainty surrounding bioavailability and blood–brain barrier (BBB) penetration of *Astragalus*-derived phytochemicals. While certain compounds such as formononetin have been implicated in BBB-associated transport modulation through LRP1 and RAGE pathways, systematic pharmacokinetic validation remains limited. For many compounds, the extent to which effective concentrations observed in vitro correspond to achievable CNS exposure in vivo remains unclear.

In addition, dosing paradigms varied widely across the included studies, with inconsistent reporting of administration routes, treatment duration, and dose justification. Such variability limits reproducibility and makes it difficult to establish dose-response relationships or therapeutic windows. These challenges are amplified in studies using crude extracts or polysaccharide mixtures, where active component concentrations are often unknown.

Another key translational limitation involves the use of experimental AD models that do not fully capture the heterogeneity of human AD pathology. Many studies relied on Aβ-induced neurotoxicity models, which are useful for mechanistic screening but may not reflect disease progression driven by complex metabolic, vascular, immune, and tau-related processes. Therefore, while *Astragalus* spp. phytochemicals demonstrate broad neuroprotective effects, their true therapeutic relevance will depend on validation in models incorporating multi-factorial pathology and clinically relevant endpoints.

### 4.3 *Astragalus* spp. in multi-herb formulations: synergy vs attribution problem

A significant proportion of included studies evaluated *Astragalus* spp. as part of complex TCM formulations or multi-herb decoctions, highlighting its ethnopharmacological relevance but also introducing challenges in mechanistic attribution. Formulations such as Danggui Buxue Tang, Jia-Wei-Kai-Xin-San, and LongShengZhi Capsule demonstrated strong neuroprotective effects through combined modulation of neurotrophic signaling, inflammatory pathways, and amyloidogenic processing. These findings support the possibility of synergistic interactions among phytochemicals derived from multiple botanical sources.

However, such synergy also complicates identification of *Astragalus*-specific contributions. In many cases, it remains unclear whether *Astragalus* spp. acts as the primary pharmacological driver or as a potentiating component that enhances the activity or bioavailability of other herbal constituents. Additionally, the absence of standardized phytochemical profiling in multi-herb preparations limits reproducibility and comparability across studies.

From an ethnopharmacological perspective, these findings remain highly relevant, as traditional formulations are typically prescribed as integrated systems rather than isolated compounds. Nevertheless, future translational research would benefit from component-deconvolution approaches, such as subtractive formulation testing, marker-compound quantification, and systems pharmacology modeling, to clarify the mechanistic contribution of *Astragalus* spp. within multi-herb therapeutic networks.

### 4.4 Limitations and methodological considerations

Several limitations should be considered when interpreting the findings of this review. First, the evidence base remains predominantly preclinical, consisting mainly of in vitro and animal studies, with limited clinical investigation. As a result, the therapeutic implications of *Astragalus* spp. phytochemicals for human AD remain speculative. Second, heterogeneity in extract composition, purification methods, dosing strategies, and outcome measures reduced comparability across studies and prevented quantitative meta-analysis.

The search strategy was restricted to PubMed and ScienceDirect due to feasibility constraints and access limitations for subscription-based databases. To compensate for this limitation, extensive citation-network expansion was conducted using ResearchRabbit, enabling identification of additional relevant studies that were not captured by the primary keyword-based search. Nonetheless, the possibility of missing eligible studies cannot be excluded, particularly non-English literature and regional databases that may contain ethnopharmacological research on *Astragalus* spp.

Additionally, formal risk-of-bias scoring tools were not applied due to substantial heterogeneity in study designs, which ranged from cell-based mechanistic studies to animal behavioral models and network pharmacology investigations. Instead, bias-related concerns such as limited blinding, inconsistent randomization reporting, and extract standardization issues were summarized narratively. These methodological factors should be considered when interpreting mechanistic conclusions and translational potential.

### 4.5 Future directions

Future research should prioritize standardized phytochemical characterization and translational validation of *Astragalus* spp.-derived compounds. Rigorous analytical profiling (e.g., LC-MS/MS or HPLC quantification) should be incorporated to ensure reproducibility and enable meaningful cross-study comparison. Studies should also report purity, marker-compound concentrations, and batch characteristics, particularly when using crude extracts or polysaccharide fractions. Future studies should, thus, adopt standardized phytochemical characterization frameworks, such as ConPhyMP, to enhance reproducibility and comparability across studies involving complex botanical extracts.

In vivo investigations should increasingly incorporate pharmacokinetic evaluation, BBB permeability assessment, and dose-response analysis to establish clinically relevant exposure profiles. Given the multifactorial nature of AD, future studies may also benefit from evaluating *Astragalus*-derived phytochemicals in models representing diverse etiologies, including metabolic dysfunction, vascular impairment, neuroimmune dysregulation, and tau pathology.

Finally, well-designed human clinical trials are essential to establish efficacy and safety. The emerging clinical interest in *Astragalus* spp., as reflected by recent trial protocols, represents a promising step toward translation. Integrating traditional knowledge with mechanistic neuroscience offers a promising pathway for the development of multi-target, plant-based interventions in Alzheimer’s disease.

## CRediT authorship contribution statement

**Yashvi Bhatt:** Writing – review & editing, Writing – original draft, Visualization, Validation, Supervision, Software, Resources, Project administration, Methodology, Investigation, Funding acquisition, Formal analysis, Data curation, Conceptualization.

## Declaration of Generative AI and AI-assisted technologies in the writing process

During the preparation of this work the author used ChatGPT (model 5.2) to assist with language refinement. After using this tool/service, the author reviewed and edited the content as needed and takes full responsibility for the content of the published article.

## Funding statement

This research did not receive any specific grant from funding agencies in the public, commercial, or not-for-profit sector.

## Conflicts of Interest

The authors declare that they have no known competing financial interests or personal relationships that could have appeared to influence the work reported in this paper.

## Footnotes

Appendix A Supplementary data associated with this article can be found in the online version at doi:10.1016/j.ibneur.2026.07.015.
