# Supplementary material for: Mechanistic convergence of Astragalus-derived phytochemicals in Alzheimer’s disease: A systematic review
Source: IBRO Neurosci Rep. 2026 Jul 31;21:450–63. doi: 10.1016/j.ibneur.2026.07.015 (PMC13471358; doi:10.1016/j.ibneur.2026.07.015)
Supplement: Supplementary file 1 [file mmc1.docx]

# Supplementary Table 1. Summary of Evidence on Astragalus Phytochemicals and Extracts in Alzheimer’s Disease

| Phytochemical(s)/Extract(s) | Model Type(s) | Mechanism(s) of action | Therapeutic outcome(s) | Citation |
| --- | --- | --- | --- | --- |
| Astragaloside IV | SK-N-SH cells | Inhibition of mPTP opening; ↓ ROS; preservation of mitochondrial membrane potential; ↓ cytochrome c release | ↓ Apoptosis; neuroprotection against Aβ1–42 toxicity | (Sun et al., 2014) |
| Astragaloside IV | chronic cerebral hypoperfusion rat models | ↓ Neuroinflammation (↓ microglial activation, cytokines); ↓ oxidative stress; anti-apoptotic (↑ Bcl-2, ↓ Bax); preservation of hippocampal neurons | Improved learning & memory; attenuation of cognitive deficits after chronic cerebral hypoperfusion | (Kim et al., 2015) |
| Astragaloside IV | “NSCs from the hippocampus of E14 rat embryos, treated the cells with ASI, and then transplanted the cells into the hippocampus of rat AD models” | ↑ Neural stem cell survival & differentiation; ↓ apoptosis; ↓ neuroinflammation; ↑ neurotrophic support (e.g., BDNF-like effects) | Enhanced efficacy of NSC transplantation; improved cognitive function; increased neuronal regeneration in AD model | (Haiyan et al., 2016) |
| Astragaloside IV | BV-2 microglia and EAE mice | Activation of glucocorticoid receptor signaling; inhibition of microglial activation; ↓ pro-inflammatory cytokines (e.g., TNF-α, IL-1β); suppression of NF-κB pathway | Anti-neuroinflammatory effect; reduced neurotoxicity; neuroprotection in inflammatory CNS conditions | (Liu et al., 2016) |
| Astragaloside IV | Aβ25‑35‑treated PC12 cells | ↓ ER stress; inhibition of p38 MAPK signaling; ↓ CHOP/caspase-mediated apoptosis | ↓ Neuronal apoptosis; protection against Aβ25–35-induced cytotoxicity | (Ma & Xiong, 2019) |
| Astragaloside IV | BV-2 cells | Promotion of microglial M2 polarization; suppression of M1 phenotype; ↓ pro-inflammatory mediators; ↑ anti-inflammatory factors | Reduced microglia-mediated neurotoxicity; enhanced neuronal survival; anti-neuroinflammatory neuroprotection | (J. Yu et al., 2019) |
| Astragaloside IV | APP/PS1 mice | ↓ Aβ deposition; ↓ neuroinflammation; ↓ oxidative stress; modulation of synaptic plasticity–related pathways | Improved cognitive function; attenuation of Alzheimer’s-like pathology in APP/PS1 mice | (W. Yu et al., 2019) |
| Astragaloside IV | Male ICR mice weighing 22-25 g (β model) | Inhibition of microglial activation; ↓ NADPH oxidase expression → ↓ ROS; ↓ neuroinflammation | Improved cognitive function; reduced neuroinflammatory damage in Aβ-induced AD model | (Chen et al., 2021) |
| Astragaloside IV | Male C57BL/6 mice (5-6 weeks old, 20-25 g) | ↓ Aβ production/aggregation; ↓ oxidative stress; ↓ neuroinflammation; modulation of apoptosis pathways; improvement of mitochondrial function | Amelioration of AD-like pathology; improved cognitive performance; reduced neuronal loss | (Wang et al., 2021) |
| Astragaloside IV | Male Sprague-Dawley (SD) rats | ↓ oxidative stress; ↓ neuroinflammation; inhibition of Aβ1–42-induced neuronal damage | Improved cognitive function; neuroprotection against Aβ1–42 toxicity | (Yanfang et al., 2024) |
| Formononetin | LPS-stimulated BV2 microglia | ↓ Microglial activation; ↓ pro-inflammatory cytokines (TNF-α, IL-1β, NO); inhibition of NF-κB signaling; ↑ ERβ expression | Anti-neuroinflammatory effect; reduced microglia-mediated neurotoxicity | (El-Bakoush & Olajide, 2018) |
|  | HT22 neurons | Reduction in pro-inflammatory cytokines | ↓ microglial toxicity |  |
| Formononetin | APP/PS1 mice | Suppressed Aβ generation via APP processing inhibition, enhanced LRP1-mediated Aβ clearance | ↓ structural damage in hippocampal vascular endothelial cells, ↓ RAGE-dependent inflammation, ↑learning, and ↑ memory | (Fei et al., 2018) |
| Caffeic Acid Phenethyl Ester (CAPE) | Male C57Bl/6 (9 weeks old, 25–30 g) mice | Activation of Nrf2/HO-1 pathway; ↑ antioxidant defense; ↓ oxidative stress; anti-inflammatory effects | Neuroprotection; reduced AD pathology; improved cognitive function | (Morroni et al., 2018) |
| Coumestrol | - | Inhibition of acetylcholinesterase & butyrylcholinesterase; inhibition of carbonic anhydrase; antioxidant activity (free radical scavenging) | Improved cholinergic neurotransmission (cognitive support); potential anti-Alzheimer’s effect; reduced oxidative stress | (Durmaz et al., 2022) |
| Kaempferol | SH-SY5Y cells and NFκB luciferase reporter cells | Inhibition of NF-κB signaling; ↓ β-/γ-secretase activity → ↓ Aβ production | Reduced Aβ levels; potential disease-modifying effect in AD | (Paris et al., 2011) |
| Kaempferol | LPS-stimulated BV2 microglial cells | Modulation of MAPK pathways (↓ p38/JNK); activation of PI3K/Akt signaling; inhibition of NF-κB; ↓ pro-inflammatory mediators (NO, TNF-α, IL-1β) | Anti-neuroinflammatory effect; reduced microglial activation; neuroprotection | (Park et al., 2011) |
| Unspecified | 12-month male mice | Anti-apoptotic effects (↑ Bcl-2, ↓ Bax); ↓ glucocorticoid-induced neurotoxicity; ↓ oxidative stress | Improved learning & memory; reduced neuronal apoptosis; neuroprotection against glucocorticoid-induced damage | (W.-Z. Li et al., 2011) |
| Unspecified | 10-week HFSTZ-APP/PS1 mice | ↓ Neuroinflammation; ↓ metabolic stress (obesity, hepatic steatosis); modulation of inflammatory signaling (without affecting Aβ deposition) | Improved cognitive function; reduced neuroinflammation; systemic metabolic improvement in AD model | (Huang et al., 2017) |
| Unspecified | APPswe/ PS1ΔE9 transgenic mice | ↓ Iron overload; regulation of iron metabolism; ↓ oxidative stress (iron-induced ROS); neuroprotective synergy of compounds | Reduced iron-induced neurotoxicity; attenuation of AD pathology; improved neuronal survival | (Y. Zhang et al., 2018) |
| Unspecified | PC12 cells | ↓ Matrix metalloproteinases (MMPs); modulation of extracellular matrix remodeling; ↓ neuroinflammation | Stabilization of blood–brain barrier; reduced neuroinflammatory damage; potential slowing of AD progression | (Z. Li et al., 2022) |
| Unspecified | In vitro enzyme inhibition assays (AChE, BChE, carbonic anhydrase, α-glycosidase) + LC-MS/MS phytochemical analysis (no in vivo AD model) | Inhibition of acetylcholinesterase & butyrylcholinesterase; inhibition of carbonic anhydrase; antioxidant activity (phenolic compounds) | Cognitive support (anti-Alzheimer’s potential); reduced oxidative stress; potential antidiabetic and antiglaucoma effects | (Güven et al., 2023) |
| Unspecified | Patients (aged 50-85) with mild to moderate AD and orthostatic hypotension | Not directly studied (clinical trial protocol; mechanisms inferred—likely anti-inflammatory, antioxidant, neuroprotective) | To be determined (aim: improved cognition, safety, and response predictors in mild–moderate AD patients) | (Cheng et al., 2024) |
| Unspecified | Aβ25–35–treated neuronal cells | Activation of Nrf2/HO-1 pathway; activation of AKT/CREB/BDNF signaling; ↓ oxidative stress | Neuroprotection against Aβ25–35 toxicity; enhanced neuronal survival; support of neurotrophic signaling | (Ji et al., 2024) |
| Unspecified | Molecular docking and network pharmacology | Inhibition of acetylcholinesterase & butyrylcholinesterase; multi-target interactions (network pharmacology); potential antioxidant effects | Enhanced cholinergic neurotransmission (cognitive support); potential anti-Alzheimer’s effect | (Lekmine et al., 2024) |
| Unspecified | Network pharmacology and molecular docking | Multi-target effects (network pharmacology): modulation of Aβ metabolism, ↓ neuroinflammation, ↓ oxidative stress, regulation of apoptosis pathways; involvement of pathways like PI3K/Akt, MAPK | Potential anti-Alzheimer’s effects (multi-pathway neuroprotection; cognitive improvement inferred) | (Lv et al., 2024) |
| Astragaloside IV from a mixture of active compounds of Epimedium, Astragalus and Radix Puerariae | APP/PS1 transgenic mouse model | ↓ Brain iron accumulation; regulation of iron metabolism; ↓ iron-induced oxidative stress | Reduced iron-mediated neurotoxicity; neuroprotection; attenuation of AD-like pathology | (Dong et al., 2015) |
| Danggui Buxue Tang containing Astragali Radix and Angelicae Sinensis Radix | Human neuroblastoma SH-SY5Y cells | ↑ Neurotrophic factor expression (e.g., NGF, BDNF); activation of neurotrophic signaling pathways | Enhanced neuronal survival & differentiation; neuroprotective effects | (A. G. W. Gong et al., 2017) |
| Danggui Buxue Tang containing Astragali Radix and Angelicae Sinensis Radix | Primary cortical neurons exposed to β-amyloid (Aβ)-induced toxicity | ↓ Aβ-induced cytotoxicity; ↓ oxidative stress; anti-apoptotic effects; modulation of neurotrophic signaling | Reduced neuronal cell death; neuroprotection against Aβ toxicity | (G. Gong et al., 2019) |
| LongShengZhi capsule containing Hirudo, Astragali Radix, Carthami Flos (Honghua), Persicae Semen (Taoren), Acori Tatarinowii Rhizoma (Shichangpu), and Acanthopanax Senticosus (Ciwujia) | APP/PS1 double transgenic mice | ↓ Oxidative stress; ↓ neuroinflammation; ↓ Aβ deposition; modulation of apoptotic pathways | Attenuation of AD-like pathology; improved cognitive function; reduced neuronal damage | (Yin et al., 2020) |
| Jia-WeiKai-Xin-San (JWKXS) containing Ginseng quinquefolium (L.) Alph.Wood (Renshen), Polygala tenuifolia Willd (Yuanzhi), Acorus tatarinowii Schott (Shichangpu), and Poria cocos (Schw.) Wolf (fuling) in a ratio of 3 : 2 : 2 : 3 | SAMP8 mouse model | ↓ Neuroinflammation; ↓ pro-inflammatory cytokines; anti-apoptotic effects (↑ Bcl-2, ↓ Bax/caspases); modulation of signaling pathways (e.g., NF-κB-related) | Improved cognitive function; attenuation of mild cognitive impairment; reduced neuronal loss | (X. Zhang et al., 2023) |
